# Genetic Diversity Increases Insect Herbivory on Oak Saplings

**DOI:** 10.1371/journal.pone.0044247

**Published:** 2012-08-28

**Authors:** Bastien Castagneyrol, Lélia Lagache, Brice Giffard, Antoine Kremer, Hervé Jactel

**Affiliations:** 1 University Bordeaux, BIOGECO, UMR1202, Talence, France; 2 INRA, BIOGECO, UMR1202, Cestas, France; Duke University, United States of America

## Abstract

A growing body of evidence from community genetics studies suggests that ecosystem functions supported by plant species richness can also be provided by genetic diversity within plant species. This is not yet true for the diversity-resistance relationship as it is still unclear whether damage by insect herbivores responds to genetic diversity in host plant populations. We developed a manipulative field experiment based on a synthetic community approach, with 15 mixtures of one to four oak (*Quercus robur*) half-sib families. We quantified genetic diversity at the plot level by genotyping all oak saplings and assessed overall damage caused by ectophagous and endophagous herbivores along a gradient of increasing genetic diversity. Damage due to ectophagous herbivores increased with the genetic diversity in oak sapling populations as a result of higher levels of damage in mixtures than in monocultures for all families (complementarity effect) rather than because of the presence of more susceptible oak genotypes in mixtures (selection effect). Assemblages of different oak genotypes would benefit polyphagous herbivores via improved host patch location, spill over among neighbouring saplings and diet mixing. By contrast, genetic diversity was a poor predictor of the abundance of endophagous herbivores, which increased with individual sapling apparency. Plant genetic diversity may not provide sufficient functional contrast to prevent tree sapling colonization by specialist herbivores while enhancing the foraging of generalist herbivores. Long term studies are nevertheless required to test whether the effect of genetic diversity on herbivory change with the ontogeny of trees and local adaptation of specialist herbivores.

## Introduction

Over the last decades, the role that biodiversity plays in ecosystem functioning has emerged as a key issue in ecology [1], [2], [3]. Although a majority of studies have focussed on the effect of plant diversity on primary production [4], a growing attention is being paid on other ecosystem services provided by biodiversity such as pest regulation.

The diversity – resistance hypothesis states that species rich plant communities suffer less feeding damage by herbivores than plant monocultures [5], [6], [7]. However two opposite effects of plant diversity on herbivory have been observed [8]. A given focal plant species can experience more damage when associated with other plant species that are more attractive or palatable for herbivores [8], [9]. This pattern is known as associational susceptibility and seems to mainly involve generalist herbivore species [7]. Conversely, a focal plant species can have less herbivore damage (*i.e.* associational resistance) when the presence of non conspecific neighbours (i) reduces host plants concentration and the probability to be located by specialist herbivores [10]; (ii) provides physical or chemical barriers to host colonisation [11], [12], [13] and (iii) increases the abundance, the diversity and/or the efficiency of natural enemies [14], [15], [16], [17]. Several meta-analyses have shown that associational resistance is more frequent than associational susceptibility but the balance between these two mechanisms is likely to depend on the identity of host plant species, herbivore feeding guilds or the way herbivory is assessed (abundance of herbivores *vs* biomass removed) [5], [7], [8], [18].

Intraspecific diversity (*i.e.* genetic diversity) is a key component of biodiversity. Recent research in the field of community genetics has shown that host plant genotype is one of the ecological filters shaping the structure of insect species assemblages [19], [20], [21] and that insect species diversity increases with the genetic diversity in host plant populations [22], [23], [24]. The question of the effects of genetic diversity on ecosystem functioning has also attracted considerable interest in recent years. It has been shown that most of the ecosystem functions provided by species diversity are also supported by genetic diversity, including plant productivity [25], [26] nutrient cycling [27], temporal stability [28], [29], [30] and resistance to invasion [22]. Despite the similarity between the effects of plant species and plant intraspecific diversity on ecosystem properties, the mechanisms underlying the biodiversity-ecosystem functioning relationship may be different at the two scales. For example, Cook-Patton *et al.* [25] showed that the increase in arthropod species richness with plant genetic diversity is mediated by arthropod abundance while resource specialisation is the main factor explaining the increase in arthropod species richness with plant species richness. It is therefore necessary to verify whether the ecological processes leading to associational resistance or susceptibility in plant species assemblages also apply to assemblages of plant genotypes.

Because herbivore species richness and abundance generally increase with genetic diversity in host plant populations [22], [25], [26], associational susceptibility may be more likely to occur than associational resistance in mixtures of host plant genotypes. In addition, generalist insect herbivores (such as grasshoppers and many leaf chewers) are known to develop better on plant species mixtures due to food resources complementation or toxins dilution, a phenomenon known as diet mixing [31], [32] that has been reported for mixtures of plant genotypes [33], [34]. Generalist herbivores are then expected to cause higher damage in genotype mixtures. Recent studies have shown positive [23], [35] or neutral [36] effects of host plant genetic diversity on the abundance of specialist herbivores. It is therefore still uncertain whether plant genetic diversity might have different effects on herbivores with different diet breadth or feeding behaviour [37], [38].

With a few exceptions [27], [39], [40], [41], studies on the functional consequences of genetic diversity for ecosystem functioning have focused on hybrids [20], [42], [43] or clones [23], [25], [35], [44], [45], [46]. Because these studies were designed so as to increase the contrast between plant genotypes, they may not be relevant to more complex processes occurring in more natural conditions [46]. We present here one of the first attempt to assess the effect of casual intraspecific plant diversity on natural insect herbivory. Using an experimental plantation of pedunculate oak saplings, we tested the following hypotheses: (i) the genetic diversity of young trees tends to increase insect herbivory (*i.e.* associational susceptibility) and (ii) the magnitude of the effect depends on host specialization of insect herbivores, being higher for more generalist species. To test these hypotheses we designed a common garden experiment with 90 synthetic mixtures of oak saplings composed of one to four half-sib families. We genotyped all saplings and evaluated the amount of damage caused by ectophagous insect herbivores (less specialized) and endophagous leaf miners (more specialized) on each individual sapling. We assessed the level of genetic diversity in each mixture and estimated the correlation between diversity and insect herbivory.

## Materials and Methods

No specific permits were required for the described field studies. The site on which the experimental common garden was established is owned by our institute (INRA) and is no subjected to any protection scheme. This work did not involve any endangered or protected species or area.

### Experimental design

In autumn 2007, we collected acorns from the canopy of four mature pedunculate oaks (*Quercus robur*), referred to hereafter as ‘mother trees’, sampled at random within a 10 km radius at a site 40 km south of Bordeaux (44°440 N, 00°460 W). In March 2008, we sowed the acorns at the nursery of the forest research centre of the French National Institute for Agricultural Research (INRA), to produce four half-sib families of oak seedlings. The seedlings were grown in individual pots containing peat and were treated with fungicide and insecticide during the first growing season (*i.e.* 2008), to prevent damage before planting. In March 2009, the seedlings were transplanted to a clearing surrounded by pine trees (*Pinus pinaster*) and broadleaved species (*Quercus robur, Quercus rubra* and *Betula pendula*).

Six different blocks were established, with 15 plots in every block, each plot corresponding to one of the 15 possible combinations of one (*n* = 4 plots, *i.e.* one per family), 2 (*n* = 6), 3 (*n* = 4) and 4 (*n* = 1) families per plot. Each plot contained four rows of three seedlings; the seedlings were 0.2 m apart and the plot area was 0.24 m^2^ (0.60×0.40). Within each plot, oak families were planted at equal density in a regular alternate pattern, such that seedlings from the same family were never adjacent in mixed plots. The plots were separated by a distance of 3 m and were randomly distributed within the blocks. Blocks were 14 m ×6 m in size and were located 4 m apart (Figure S1).

The experimental site was fenced to prevent grazing by mammalian herbivores. The herbaceous plants growing between plots were removed by mowing, twice yearly. Pine bark chips were spread on the soil of each plot to control the vegetation and limit evaporation. Plots were watered during the summer of 2009, to minimise seedling mortality. In August 2011, 25 out of the 1080 planted seedlings were dead (*i.e.* 1055 survived).

### Herbivory assessment

Insect herbivory was assessed by the visual inspection of 20 leaves on each four-year-old sapling, in August 2011. Five leaves were sampled at the tip and five at the base of two branches randomly chosen at the top and two branches randomly chosen towards the bottom of the sapling. We also recorded the total height of each sapling during this herbivory assessment.

Herbivore damage on oak leaves was assigned to four different trophic guilds: *chewers* (mostly adult Curculionidae or Chrysomelidae and Lepidoptera caterpillars), *skeletonisers* (adult grasshoppers and Tenthredinoidea larvae), *rollers* (mostly Lepidoptera larvae) and *miners* (mostly Microlepidoptera larvae). No gall makers were observed. The percentage leaf area affected was visually estimated for each leaf and each guild using six classes (0%, 1–5%, 6–15%, 16–25%, 26–50%, 51–75% and >76%) and then averaged per sapling.

Damage due to skeletonisers and leaf-rollers were very rare. We therefore pooled these two guilds with the chewers and classified the damage inflicted as being due to ‘ectophagous insects’. Previous work by Giffard *et al.* [47] in the same study area showed that most of ectophagous insect herbivores found feeding on *Q. robur* are polyphagous species able to consume plant tissues from different genera and families and may be then considered as generalists (see [47] for a list of the commonest species). Leaf miners are different from the other insect herbivores found on oak saplings in that they are endophagous and much more specialized (they develop on a narrow spectrum of species within the Fagaceae family). Damage by leaf miners was quite frequent but minor in term of leaf area impacted. In addition, the leaf surface affected by a mine is dependent on the timing of assessment, while the presence or absence of a mine is not. We therefore used the density of mines per sapling (number of mines/20 leaves) to quantify damage due to these specialist insects.

### Genotyping of oak saplings

All oak saplings and the four mother trees were genotyped with 12 microsatellite markers (see Guichoux *et al*. [48] for details), using one leaf per sapling and per mother tree collected in August 2010. Leaves were dried and stored separately before DNA extraction and gene amplification. We isolated DNA from five leaf discs, each 5 mm in diameter, from each sample with the Invisorb DNA plant HTS 96 kit (Invitek, Berlin, Germany). We used the 12plex SSR (Single Sequence Repeats) kit developed by Guichoux *et al.* [48] for genotyping. We scored SSR profiles, using real allele sizes and alleles were binned with the Microsoft Excel macro AUTOBIN program (available from http://www4.bordeaux-aquitaine.inra.fr/biogeco/Ressources/Logiciels/Autobin) developed by Guichoux *et al.* [49].

Among the 1059 surviving individuals in 2010, 1032 were successfully genotyped. The mean proportion of loci succesfully typed was 99.7%. The mean number of alleles per locus was 11 (range: 6–19). More detailed information about genetic structure of the oak seedlings population is provided in Table S1. Seventeen offspring (1.7%) were excluded from the analysis because their genotype at multiple loci did not match that of any mother tree. 134 offsprings showed only one mismatch with the corresponding mother tree. These offsprings were used to identify loci with genotyping errors before correction. Error rates based on these comparisons were low for 10 markers (<2%), high for one single-nucleotide marker (1.92% for the PIE258 marker) and high for another marker (10.49% for the PIE020 marker). Comparisons of the genotypes of mother trees and offspring revealed that manual binning was incorrect for the single-nucleotide marker and a null allele in the offspring of one mother tree, for the PIE020 marker. Single-nucleotide errors were corrected for further analysis and manual binning was repeated for the PIE258 marker. The PIE020 marker was removed from the data set. We finally retained 11 markers for the genotyping of 1016 offsprings plus the four mother trees.

### Estimation of genetic diversity

We initially used the number of maternal lineages per plot as a measure of genetic diversity. However, as a given mother tree could have been pollinated by several father trees, the offspring may be half-sibs or full-sibs and the proportion of the two types of saplings could vary within families and within sapling assemblages. The number of maternal lineages per plot may therefore underestimate genetic diversity and be poorly correlated with variation in insect damage.

We then determined SSR genotypes, to calculate the genetic relatedness between oak saplings, thereby improving estimates of genetic diversity per plot and switching from an almost categorical (1, 2, 3 or 4 maternal lineages per plot) to a more continuous (90 individual scores of genetic diversity) variable. Hereafter, *genetic diversity* (GD) refers to the number of maternal lineages per plot, whereas *genetic relatedness* (GR) refers to the mean between-saplings relatedness per plot.

Genetic relatedness was calculated with CoAncestry software [50]. We used the DyadML estimator (a dyadic likelihood estimator described in [51]) because the simulated values of relatedness it provided were the closest to expected values (*i.e.* 0.5 for full sibs, 0.25 for half sibs and 0 for unrelated saplings). GR was calculated for all pairs of individuals (*n* = ½(1016×(1016–1))  = 515,620 pairs) and we used these values to calculate a mean genetic relatedness for each plot. Mean genetic relatedness significantly differed between plots with different numbers of maternal lineages (Kruskal-Wallis test: *K_calc_*  = 845.55, *df*  = 3, *p*<0.001), decreasing with increasing number of lineages (Figure S2). However, genetic relatedness also varied considerably within each level of genetic diversity, thus supporting the use of the two indices. As they were highly correlated (*r* = –0.80, Figure S2), GD and GR were introduced separately in further models.

Owing to missing genotypes (dead saplings, unamplified DNA, mismatch between observed genotype and mother tree), mean relatedness was averaged across a variable number of individual saplings per plot (9 to 12). For the sake of consistency, missing genotypes were also removed before the analysis of insect damage data. The final dataset contains 1002 individuals (6 blocks ×15 plots ×12 trees –25 dead saplings –53 unamplified or mismatched genotypes).

### Statistical analyses

Response variables (*i.e.* herbivory by ectophagous insects, abundance of leaf miners and sapling height) were analysed using each individual tree as a replicate to make it possible to test for the possible effects of interactions between mother tree identity (MT) and GD or GR. We accounted for spatial replication by nesting the ‘population effect’ (*i.e.* 1 population  = 1 plot  = 12 saplings) within the block effect, both factors being treated as random effects in all mixed models, in order to specify that individual observations were correlated within blocks and within plots.

We first tested the MT effect on response variables in monocultures alone, to avoid confounding factors. Mother tree identity was part of the experimental design and we were interested in its influence on the mean of herbivory by ectophagous insects, abundance of leaf miners and tree height. We therefore treated this factor as a fixed effect because there were not enough levels on which to base an estimate of the variance of the total population (only four different mother trees).

In order to determine potential genetic effects on sapling height, we first performed two sets of linear mixed models with MT and GD or GR, separately, and their interactions as fixed effects. We then carried out linear mixed models to test the effect of sapling height, MT, GD or GR, and their interactions on herbivory by ectophagous insects (% leaf area damaged) and by endophagous insects (abundance of leaf miners), separately. Prior to analyses, continuous explanatory variables (GD, GR and sapling height) were centred (*i.e.* subtracting the sample mean from all observations) and reduced (*i.e.* dividing centred variables by their sample standard deviation) in order to make model coefficients comparable within and between models [52] and to allow estimating the magnitude of effects. Centring variables also makes main effects biologically interpretable even when involved in interactions [52].

In all mixed models, we applied a model simplification procedure and reduced each maximal mixed model by removing non significant interaction terms, starting with the highest order interaction, to finally retain the least parameterized models including only simple terms and significant interaction terms.

Test statistics for fixed effects were based on F values for linear mixed models (herbivory by ectophagous insects and sapling height) and on χ^2^ values (loglikelihood ratio tests with one degree of freedom) for generalised linear mixed models performed on the abundance of leaf miners. Log-likelihood R^2^ values were calculated to estimate the amount of variance explained by each independent variable [53].

Data for sapling height and damage due to ectophagous insects were analysed with linear mixed models with the *lme* procedure [54] in R [55]. Tree height was square-transformed and percentage data were transformed with the *logit* function [56] to meet the assumptions of these tests, which were checked by graphical analyses and Shapiro-Wilk tests on model residuals. The abundance of leaf miners per tree was expressed as counts, which were analysed with generalized linear mixed models by specifying a Poisson error structure, with the *lmer* procedure in the lme4 package in R [57].

We used the method developed by Loreau and Hector [58] and adapted by Unsicker *et al.* [1] to quantify the net genetic diversity effect on herbivore damage. We first calculated the observed relative forage of the family *i* (*RF*_O*i*_) as the ratio of the damage observed on each family *i* (*i* from 1 to 4) in a mixture (*C_i_*) to that observed on this family in monoculture (*M_i_*) [1]:
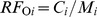
(1)


The expected relative forage of the family *i* (*RF*_E*i*_) under the null hypothesis (*i.e.* no effect of genetic diversity on damage) was simply its proportion in the mixture, *i.e.* 1/*n* where *n* is the number of families in the mixture [1], [58].

The deviation of the observed relative damage in a mixture from the relative damage expected in the corresponding monoculture was thus:



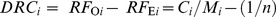
(2)


The total observed damage in the mixture was calculated as:



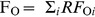
(3)


The total expected damage in the mixture was calculated as:



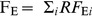
(4)


A positive NGDE indicates associational susceptibility (higher level of damage observed in mixtures than expected from mean damage levels in the corresponding monocultures), whereas a negative NGDE indicates associational resistance (lower level of damage observed in mixtures than expected from mean damage levels in the corresponding monocultures).

The NGDE can be further divided into two additive components: a complementarity effect (CE) and a selection effect (SE) [1], [58].



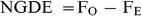
(5)


The CE is assessed by calculating the mean *ΔRC_i_* over all families at the plot level:
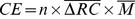
(6)


CE measures the change in mean relative forage of the species. CE is positive when the mean relative forage increases *i.e.* when oak families are, on average, consumed more in mixtures than it would be expected from their consumption in monocultures.

The calculation of SE takes into account the covariance between ΔRC_i_ and M_i_:



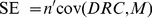
(7)


SE values are used to determine whether there is a relationship between consumption in the monoculture and relative forage in mixtures. SE is positive when plant species that are consumed in larger amounts in monocultures (less resistant) also have higher relative forage values in mixtures, thus making a greater contribution to total plot damage.

NGDE, CE and SE were calculated for all levels and combinations of mixtures within each block, giving a total of 66 comparisons between observed and expected values. The significance of each effect (NGDE, CE, SE) was determined by one sided t-tests [58]. We first tested grand mean values across all mixtures against zero, to determine whether they differed significantly from the weighted average of the response variable in monocultures. We also assessed the significance of the NGDE, CE and SE against zero for each level of genetic diversity. We used analyses of variance to assess change in these three effects along the gradient of GD [58].

## Results

### Effects of genetic diversity and relatedness on sapling height

Mean sapling height significantly differed between oak families (*F*_3,909_  = 2.88, *p* = 0.035) but we observed no significant effect of genetic diversity (GD: *F*_1,83_  = 0.02, *p* = 0.880) or genetic relatedness (GR: *F*_1,83_ <0.01, *p* = 0.984) on sapling height.

### Effects of genetic diversity and relatedness on insect herbivory

**Figure 1:**
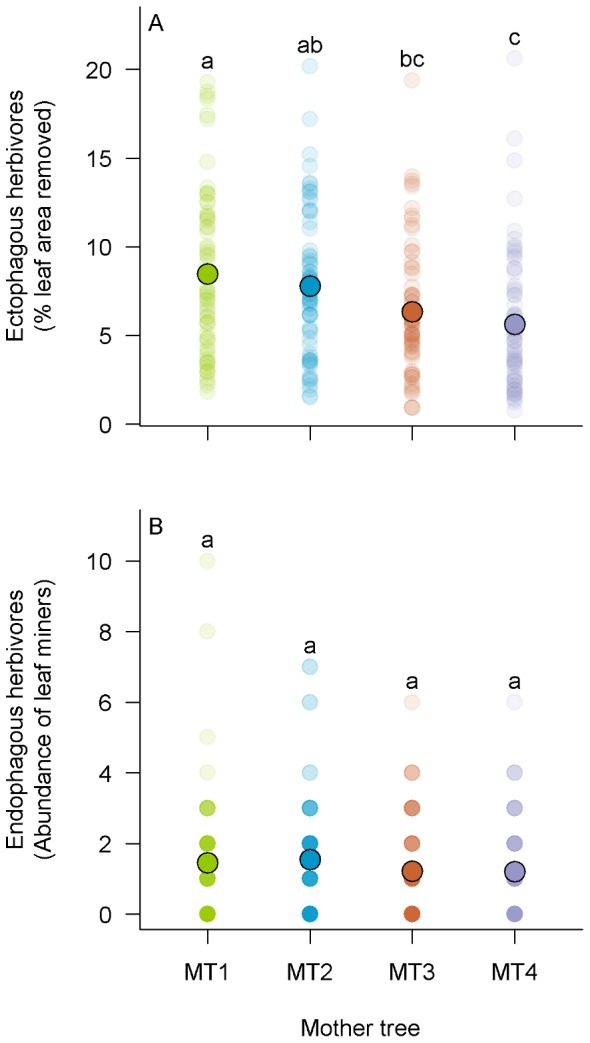
Effect of mother tree identity on insect herbivores in monocultures. (A) Effect of mother tree identity on damage (% leaf area removed) due to ectophagous herbivores. (B) Effect of mother tree identity on the abundance of endophagous insect herbivores. Semi transparent coloured circles represent individual saplings. Darkest circles represent overlapping datapoints. Solid black circled dots indicate the mean values in monocultures for all saplings and all blocks. Same letter above two lines of dots indicates that the corresponding means were not significantly different (LMM and GLMM on monoculture plots).

**Figure 2:**
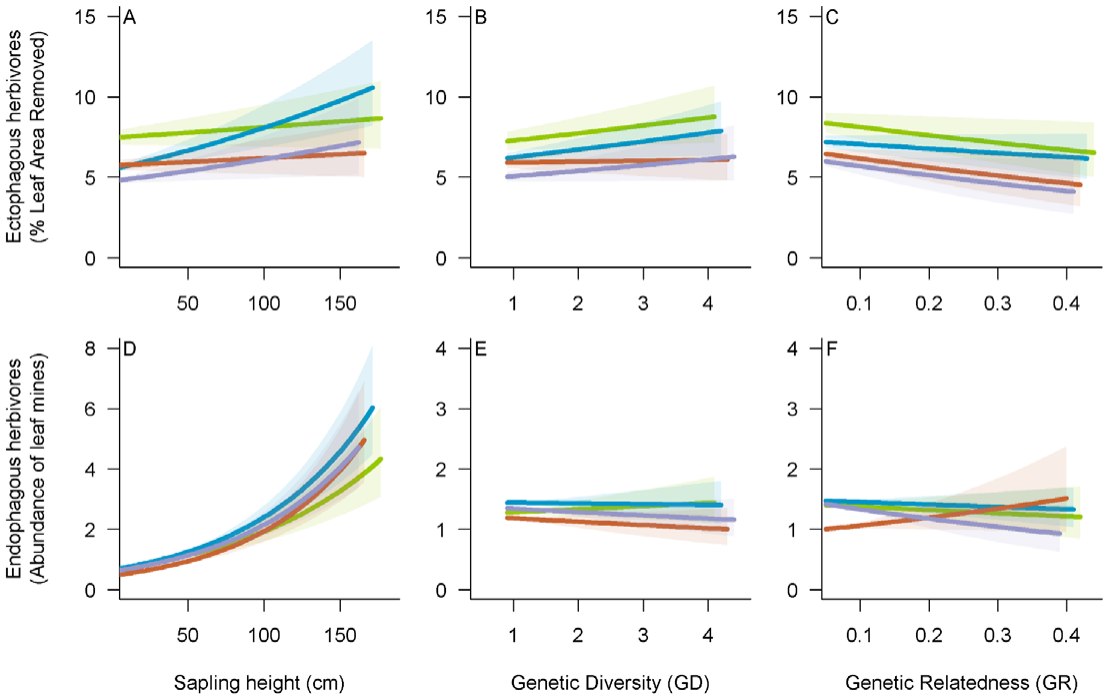
Effects of sapling height, genetic diversity and genetic relatedness on ectophagous and endophagous insects. Effects of sapling height (A, D), genetic diversity (B, E) and genetic relatedness (C, F) on damage due to ectophagous insects (A, B, C) and on the abundance of endophagous insects (D, E, F). The different colours indicate regression lines for different families (MT1: green, MT2: blue, MT3: red, MT4, purple). The shaded areas indicate the corresponding 95% confidence intervals.

Damage due to ectophagous insects was significantly affected by MT, GD, GR and sapling height (H), but not by interactions between these factors (Table 1). Significant differences in damage levels between families were observed in monocultures (on average 5.5 and 8.3% of leaf area was removed in the more and the less resistant families, respectively), suggesting a genetic control of oak saplings resistance to ectophagous insects (*F*_15,244_  = 4.81; *P* = 0.015; *R* ^2^ = 0.04, Figure 1A). Damage also increased significantly with the GD of saplings (Figure 2B) and decreased significantly with increasing GR, regardless of the family considered (Figure 2C), indicating that the presence of more genetically diverse neighbours increased the risk of damage and that this risk increased with the diversity of conspecific neighbours. However, the magnitude of this effect was low, leaf area removed being on average 6.9% in monocultures and 7.9% in 4-families mixtures. Damage by ectophagous insects also increased significantly with sapling height (Table 1, Figure 2A). The effects of GD, GR and H on damage by ectophagous herbivores were comparable in terms of magnitude, as shown by standardized model coefficients (Table 1). The effects of GD and GR on herbivory seemed to be direct rather than mediated by the genetic control of sapling height as (i) GD and GR had no effect on height and (ii) MT×H (*F*_3,905_  = 1.56; *p-value*  = 0.198), GD×H (*F*_1,901_  = 0.08; *p-value*  = 0.775) and GR×H (*F*_1,907_  = 2.92; *p-value*  = 0.088) interactions had no significant effect on damage (Table 1).

**Table 1:** Summary of the results of linear mixed models assessing the effect of sapling height (H), mother tree identity (MT), genetic diversity (GD) and genetic relatedness (GR) between oak saplings and their interactions on herbivory by ectophagous insects and on abundance of endophagous insects (leaf-miners).

		Ectophagous insects					Endophagous insects			
		*df* a	Coefficients of regression (± SE)	F-value	p-value	Log-likelihood R^2^	Coefficients of regression (± SE)	χ^2^	p-value	Log-likelihood R^2^ b
Genetic diversity	H	1, 908	0.05 ± 0.02	6.51	**0.011**	0.006	0.31 ± 0.052	127.53	**<0.001**	
	MT	3, 908		18.39	**<0.001**	0.052		7.65	0.054	
	GD	1, 83	0.06 ± 0.03	4.45	**0.038**	0.004	−0.001 ± 0.080	<0.001	0.995	
	H × GD	-					−0.003 ± 0.088	12.50	**0.014**	0.004
Genetic relatedness	H	1, 908	0.05 ± 0.02	6.57	**0.011**	0.007	0.31 ± 0.05	128.48	**<0.001**	
	MT	3, 908		19.22	**<0.001**	0.054		7.51	0.057	
	GR	1, 83	−0.06 ± 0.03	4.49	**0.037**	0.004	−0.04 ± 0.08	1.08	0.300	
	H × GR	-					0.08 ± 0.27	15.49	**0.004**	0.007

Results are given from LMM and Poisson GLMM for ectophagous and endophagous herbivores respectively.

a *df* degrees of freedom (numerator, denominator).

b Log-likelihood R^2^ were not estimated in case of significant H × GR and H × GD interactions.

For endophagous herbivores (*i.e.* leaf miners), sapling height emerged as the main factor determining their abundance on individual saplings (Table 1, Figure 2D). GD×H and GR×H interactions also significantly affected the abundance of leaf miners (Table 1) while H×MT did not (χ^2^ = 1.05; *p-value*  = 0.789). The coefficient estimate of GD×H interaction term was negative (Table 1) which means that the strength of the effect of sapling height on abundance of leaf miners decreased when increasing GD. The opposite was true for GR×H (Table 1), which is consistent with the negative covariation between GD and GR. However, standardized coefficients of regression of both GD×H and GR×H were low compared to the coefficient of regression for H. The simple effects of MT (Figure 1B), GD (Figure 2E) and GR (Figure 2F) on leaf miner abundance were not significant (Table 1).

### Net genetic diversity effect

**Figure 3:**
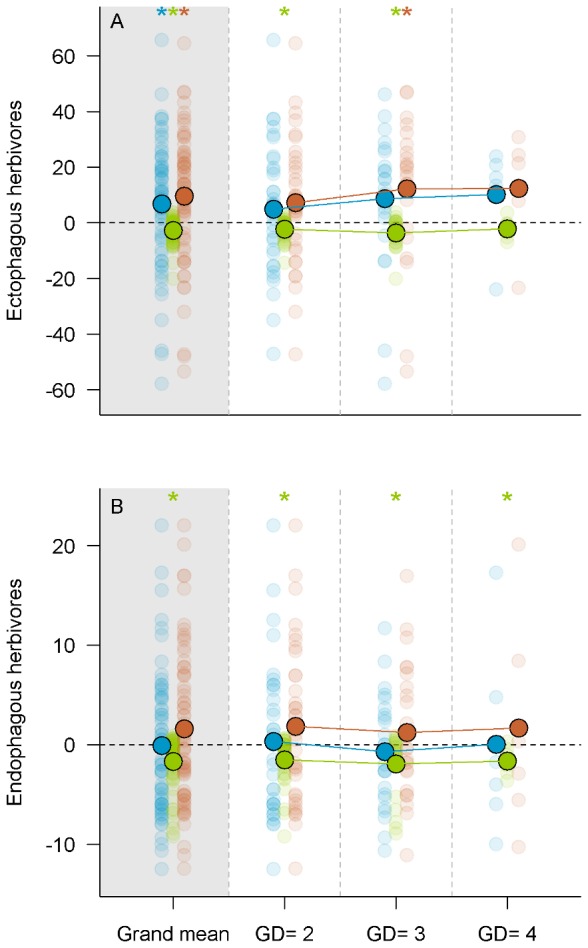
Non-additive effect of genetic diversity insect herbivores. (A) Test of the non-additive effect of genetic diversity on ectophagous insects. (B) Test of the non-additive effect of genetic diversity on endophagous insects. Semi transparent circles represent individual values per plot for net genetic diversity effect (NGDE, blue), complementarity effect (CE, red) and selection effect (SE, green). Solid black circled dots are the averaged values for all plots (grand mean) and each level of genetic diversity (GD). The ‘*’ symbol are for means value significantly different from zero.

The net genetic diversity effect (NGDE) on herbivory by ectophagous insects was overall significantly positive (Table 2), indicating a higher level of damage in mixtures than expected from monocultures (*i.e.* associational susceptibility). Both complementarity and selection effects (CE and SE) were significantly different from zero (Table 2) but had opposite signs (Figure 3): mean CE was positive and more than three times higher than mean SE, which was negative. The resulting positive NGDE was therefore principally due to the positive complementarity effect. Mean NGDE and CE were consistently positive at each level of genetic diversity, and SE was significantly negative at all but the higher level of GD (Figure 3, Table 2). A negative Selection Effect indicates a negative covariation between damage in monocultures and the deviation between observed and expected relative damage in mixtures (Figure S3). For families with lower levels of damage in monocultures (*i.e.* intrinsically more resistant), herbivory in mixtures was much higher than expected (Figure S3).

**Table 2:** Summary of *t* values from *t*-tests for net genetic diversity effect (NGDE), complementarity effect (CE) and selection effect (SE) on damage of ectophagous and abundance of endophagous (leaf-miners) insects, and on sapling height, for all mixtures (grand mean) and for each level of genetic diversity (GD).

		Grand mean	GD = 2	GD = 3	GD = 4
	*df*	65	35	23	5
Ectophagous insects	NGDE	2.44*	1.30	1.79	1.43
	CE	3.37**	1.95	2.39*	1.57
	SE	−5.51***	−4.07***	−3.51**	−1.38
Endophagous insects	NGDE	−0.08	0.28	−0.61	0.01
	CE	1.70	1.40	0.89	0.37
	SE	−5.46***	−3.96***	−3.16**	−2.69*
Sapling height	NGDE	0.57	1.01	−1.12	2.76 *
	CE	1.05	1.34	−0.77	2.90 *
	SE	−4.31***	−3.51**	−2.46*	−0.91

Significant t-values are in bold: (***) P-value <0.001, (**) 0.001< P-value <0.01, (*) 0.01< P-value <0.05.

There was no significant NGDE or CE on the abundance of endophagous herbivores (Table 2). By contrast, SE was significant and negative at all levels of genetic diversity (Figure 3, Table 2). Thus, for the families showing a tendency for higher infestation in monocultures (more susceptible), relative infestation levels were lower than expected in mixtures, the opposite being true for less susceptible families. CE and SE were of similar magnitude but of opposite signs, accounting for the null NGDE.

NGDE and CE were not significant for sapling height, for either the grand mean, or for any of the levels of genetic diversity, with exception of the 4-families mixtures (Table 2). SE was consistently and significantly negative (but for GD = 4).

## Discussion

Based on a large number of samples and a manipulative experiment this study shows for the first time that genetic diversity can trigger associational susceptibility to insect herbivory [8], [9] in tree saplings. This process describes an increase in insect herbivory with increasing genetic diversity in host population.

The relative importance of genetic diversity *vs.* other ecological factors as drivers of ecosystem processes is a central issue for community genetics [46], [59], [60]. In our study we found that the effects of genetic diversity or relatedness on insect herbivory were overall significant but low in terms of magnitude. These results are consistent with the small effects of tree genetic diversity on structuring the insect community associated with pedunculate oak, as recently reported by Tack *et al.* [39], [40]. In addition, we showed that sapling height was as important as genetic diversity for predicting generalist herbivore damage and the best predictor of endophagous herbivores abundance. These findings suggest that the influence of host tree genetic diversity on insect herbivores may originate in the variance of particular functional traits.

If genetically based differences in tree susceptibility to herbivores is now well documented [39], [61], [62], [63], [64], the effect of genetic diversity on insect damage has rarely been investigated and most often on crops or herbaceous plants [23], [36], [65]. Recently, Tack *et al.* [39], [40] studied the effects of genotype identity and diversity on the structure of endophagous insect communities on *Quercus robur*, but they did not measure corresponding herbivory. We are aware of only two studies that investigated the relationship between tree genetic diversity and insect damage. They reported a trend towards higher levels of pest damage in monocultures than in mixtures of willow clones [66], [67] but they focused on only two specialized leaf beetles. The consequences of tree genetic diversity on total herbivory remain largely unknown. A similar concern is currently emerging about the effect of plant species diversity on insect herbivory. The diversity-resistance relationship has been clearly demonstrated by meta-analyses focusing on individual species-species interactions [7], [68], [69]. However, the diet breadth of insect herbivores emerged as a key factor accounting for differences in insect response to plant diversity. Herbivory by oligophagous species is often reduced in mixed-species forests in comparison to monospecific forests, whereas the response of polyphagous insect species is more variable [7]. Several examples of such opposite patterns have recently been reported, with higher levels of damage caused by polyphagous insects [70], [71] and lower abundance of oligophagous insects [72] in more diverse plant communities. The effect of plant diversity on total insect herbivory may then primarily depend on the share of generalist and specialist herbivore species. Here we tentatively addressed this issue by considering two guilds of herbivores of contrasting diet breadth. Oak leaf-miners are oligophagous species that develop a narrow range of species within the *Quercus* and the *Castanea* genera while all the ectophagous insects we observed in the field were polyphagous species able to feed on host plants belonging to different families (see [47] for the list of insect herbivore species found on oak trees in the study area). As endophagous herbivores, leaf-miners have an intimate relationship with their host and are expected to be more dependent on host genotype than ectophagous insects that can move freely and exploit several hosts during their development [20], [46].

### Response of ectophagous herbivores

In the present study, we observed that the four oak families displayed different levels of resistance to generalist insect herbivores but also differed significantly in sapling height. Herbivory by generalist insects increased with sapling height. However there was no significant interaction between the effect of sapling height and genetic identity on damage by ectophagous herbivores. Moreover the genetic diversity and relatedness had only weak effects on sapling height whereas they significantly affected ectophagous insect herbivory. So the observed increase in damage caused by these herbivores in genetically diverse oak sapling mixtures was not mediated by differences in height. Yet, four ecological mechanisms may account for the observed relationship between genetic diversity and herbivory by ectophagous insects.

#### (i) Herbivore abundance

Herbivore abundance has been reported to increase with genetic diversity [25]. Associational susceptibility may then have been driven by an increase in abundance of generalist herbivores in genotype mixtures. However, as we did not sampled insects, we cannot validate this hypothesis. In addition, the relationship between herbivore density and herbivory damage remains unclear and we are not aware of any study that convincingly demonstrated an increase in herbivory with the abundance of herbivores.

#### (ii) Mixing diet

Herbivory by ectophagous insects increased in genotype mixtures because of a higher consumption of the four oak families, regardless their intrinsic susceptibility in monocultures, as evidenced by a significant and positive complementarity effect. This is consistent with the observation that generalist insect herbivores can increase their fitness by feeding on different host plants [1], [2]. Different plant genotypes may provide insects with feeding resources of different qualities [41]. Mixtures of genotypes are therefore likely to improve diet mixing, which is known to benefit generalist herbivores [1], [33], [34]. It has been also proposed that feeding on different host plants results in the dilution of toxic compounds present in the plant tissues, allowing a more balanced input of nutrients [31], [32]. It should be of great interest now to investigate leaf chemistry and check whether the blends of secondary metabolites involved in plant defence can explain herbivory patterns in genotypes mixtures and possibly changes with the genetic diversity of mixtures.

#### (iii) Spill over

The higher damage by herbivorous insects in plant species mixtures (*i.e.* associational susceptibility) has been initially attributed to a spill over of generalist herbivores from their preferred host plants to nearby suitable but less suitable host plants [9]. Despite the fact we did not monitor the temporal dynamic of ectophagous insects on individual oak saplings, the negative selection effect we report, though low, may account for such a spill over. Indeed, a negative selection reveals the existence of negative covariance between observed damage in mixtures and observed damage in monocultures: the increase in damage with genetic diversity was higher for families that suffered less damage when growing in monocultures. This is consistent with the hypothesis of greater colonisation through contagion, with the transfer of insects from more to less palatable families, in sapling mixtures. A similar behaviour was recently reported by Utsumi *et al.* [35] who observed a shift of insect herbivores from more to less preferred host genotypes in mixtures of annual plants.

#### (iv) Host location

The way insect herbivores perceive their host plants may change with their genetic diversity as recently suggested by Crawford *et al.* [23] who showed a non additive increase in gall abundance on patches of *Solidago altissima* with higher genetic diversity. At the plot scale, associational susceptibility may be explained by a better patch detection by foraging herbivores. For example, the mixture of sapling genotypes may have increased the probability of incorporating tall saplings that could be easier to detect and colonise, as suggested by our observation of a significant effect of sapling height on insect damage. Consistent with this hypothesis, the difference in height between taller (75^th^ percentile of heights distribution) and medium-sized (median of heights distribution) saplings tended to increase with genetic diversity at the plot level (Figure S4). In addition to visual cues that shape plant “physical” apparency, host plant location by insect herbivores is most often mediated by olfactory cues [73]. Host-plant recognition depends on ratios of plant volatiles and not just on detection of the presence or absence of particular compounds [74]. Insects use blends of volatile compounds to distinguish between host and non-host plant species. It has recently been suggested that there is redundancy in the composition of host odour blends, with some components being substitutable to others [75]. It is therefore possible that a mix of host plant genotypes is more likely to produce the right combination of attractants than a monoculture of a single plant genotype [76].

However, as we did not sample insect herbivores, it is difficult to determine which one of these mechanisms is the more likely or if they operate synergistically. For example, for a single herbivore, diet mixing might actually lead to less herbivory if that individual is able to acquire more nutrients with a variety of host genotype consumed. But on the other hand, if mixed diets are more preferable, and if mixed diets are associated with mixed host finding cues, it might attract more individuals and lead to greater overall herbivore damage. As a result, the abundance of herbivores may have ultimately been the primary driver of associational susceptibility.

### Response of endophagous herbivores

None of the genetic attributes (identity, diversity or relatedness) had a significant effect on the abundance of specialist herbivores (leaf-miners). This finding is consistent with previous studies showing that genotype [39], and genetic diversity [39] are poor predictors of the diversity of specialist herbivores (leaf-miners and gall-makers) feeding on oaks. Instead, sapling height emerged as the key determinant of leaf miner abundance. Consistently, the maternal lineages that produced the tallest saplings (MT2 and MT1) were also more infested by leaf miners (although not significantly) than those in which saplings were significantly smaller suggesting that genetically based differences in sapling height may drive differences in the abundance of leaf miners. The negative selection effect on abundance of leaf-miners indicates that the oak families that tended to be less infested in monocultures also tended to be more often colonised by leaf miners in mixtures than in monocultures. As the larval stages of leaf miners cannot relocate after oviposition and cannot shift from one host plant to the next in order to find new (spillover) or complementary (mixed diet) feeding resources, the distribution of leaf miners between and within plots thus reflects the choice of oviposition site by females. As proposed for ectophagous herbivores, a possible explanation of the negative selection effect is that mixing genotypes resulted in a greater probability of including taller and then more attractive saplings to endophagous insects (Figure S4). One cannot exclude that the combination of relevant attractants was also more likely to occur in more diverse genotypes mixtures.

The mean abundance and species richness of leaf miners were very low in our experiment, with abundance scores of 0 to 10 mines in 20 leaves per sapling and 85% of total abundance represented by only two of the nine observed species (*Phyllonorycter* sp. and *Stigmella* sp.). Separate analyses of each species of leaf miner would have generated too many zero counts, so we decided to pool data into a single category of “insect specialists”. However, Tack and Roslin [39] showed that *Phyllonorycter* sp. and *Stigmella* sp. responded differently to genetic and environmental treatments. Considering the abundance of several leaf-miner species together may therefore have prevented the detection of species-specific abundance patterns. Further investigation, after the oak saplings have been colonised by a larger number of insect species, are required for more detailed comparisons of the responses of generalist and specialist herbivores to genetic diversity.

## Conclusion

Unlike plant species richness, plant genetic diversity may not provide sufficient functional contrast to prevent host colonization by specialist herbivores while enhancing the foraging of generalist herbivores. Overall, we observed a significant effect of tree genetic diversity on generalist herbivores but not on specialists. Increasing genetic diversity resulted in higher damage by generalist herbivores because of (i) a general increase in leaf consumption in more diverse genotype mixtures (*i.e.* positive complementarity effect) and (ii) an increased damage exposure of individuals from more resistant genotypes in the vicinity of individuals from more susceptible genotypes (*i.e.* negative selection effect). To date many studies have shown that herbivore diversity increases with host plant genetic diversity [22], [23], [24], [25], [26]. There is a need now to reconcile the two approaches and investigate the relationship between the diversity of herbivores and the resulting herbivory along gradients of plant genetic diversity. In addition, when saplings develop into young trees, they may be more easily located and infested by more specialized herbivores, therefore benefiting from being part of a mixed-genotype community [66], [67]. We would therefore advocate long-term monitoring of the dynamics of sapling colonisation by insects with various degrees of host plant range limitation, to determine whether the magnitude and direction of the effect of genetic diversity on associational herbivory change with the ontogeny of focal tree species.

## Supporting Information

Figure S1 **Experimental design.** Each colored square represents an individual oak sapling.
(DOCX)

Figure S2 **Effect of the number of half-sib families per plot (Genetic Diversity, GD) on the mean genetic relatedness among oak seedlings within plots (Genetic relatedness, GR).** Open circles represent individual plots (*n* = 90); filed circles represent mean genetic relatedness per level of genetic diversity.
(DOCX)

Figure S3 **Negative selection effect of genetic diversity on exophagous herbivores.**
(DOCX)

Figure S4 **Effects of genetic diversity on oak height heterogeneity within plots.** Each dot represents the mean difference (± SE) between the 75^th^ percentile and the median (50^th^ percentile) of sapling heights distribution within plots. This difference indicates how far taller trees were apparent to herbivores within plots.
(DOCX)

Table S1 **Summary of genetic data describing the genetic structure of the population used to constructed experimental plots.**
(DOCX)
