# Supplementary material for: Genetic Diversity Increases Insect Herbivory on Oak Saplings
Source: PLoS One. 2012 Aug 28;7(8):e44247. doi: 10.1371/journal.pone.0044247 (PMC3429418; doi:10.1371/journal.pone.0044247)
Supplement: Figure S1 — Experimental design. Each colored square represents an individual oak sapling. (DOCX) [file pone.0044247.s001.docx]

**Supplementary information**


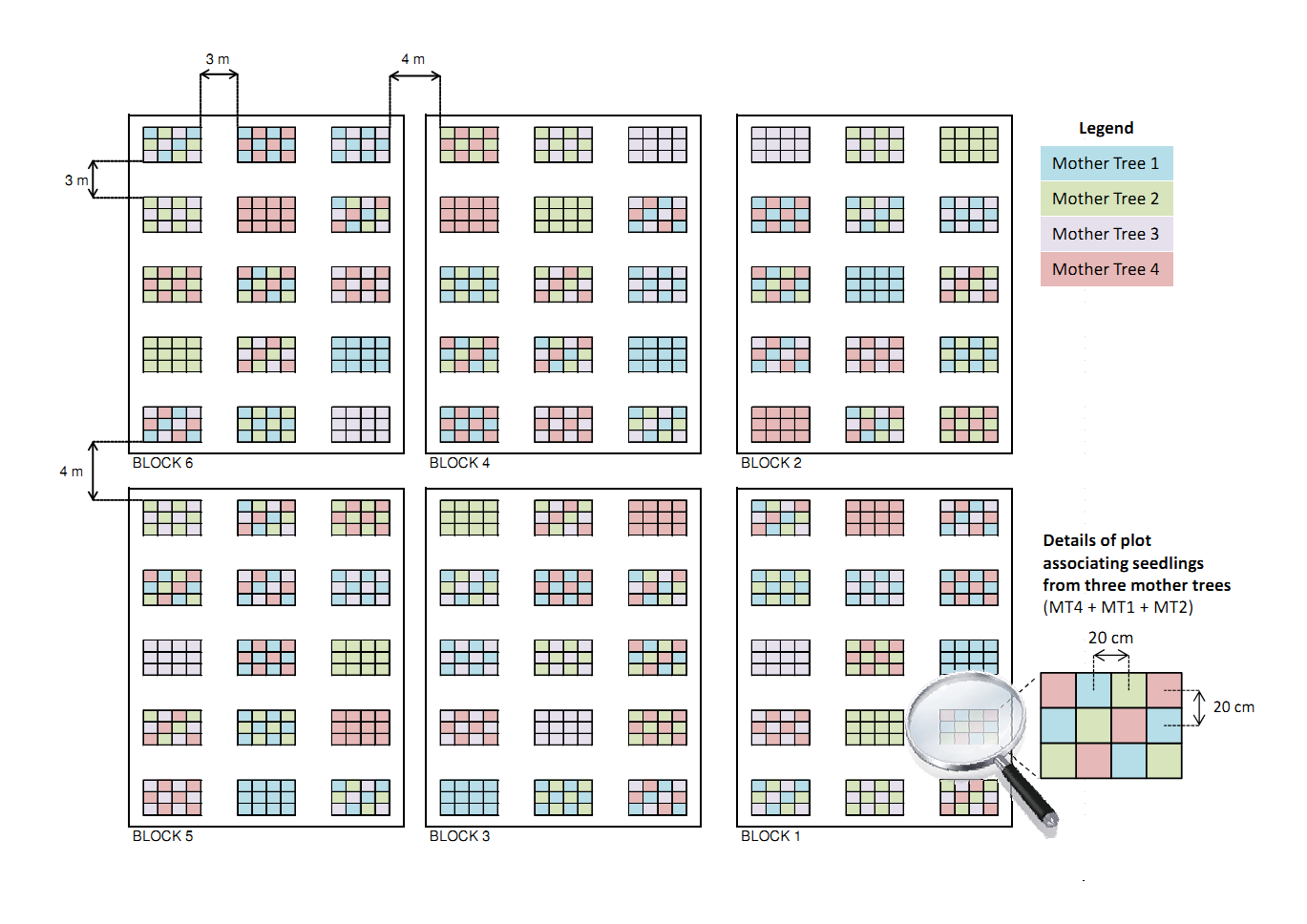


Figure SI1: Experimental design. Each colored square represents an individual oak sapling.
