# Supplementary material for: Genetic Diversity Increases Insect Herbivory on Oak Saplings
Source: PLoS One. 2012 Aug 28;7(8):e44247. doi: 10.1371/journal.pone.0044247 (PMC3429418; doi:10.1371/journal.pone.0044247)

**Supplementary information**

Figure SI2: Effect of the number of half-sib families per plot (Genetic Diversity, GD) on the mean genetic relatedness among oak seedlings within plots (Genetic relatedness, GR). Open circles represent individual plots (*n*= 90); filed circles represent mean genetic relatedness per level of genetic diversity.


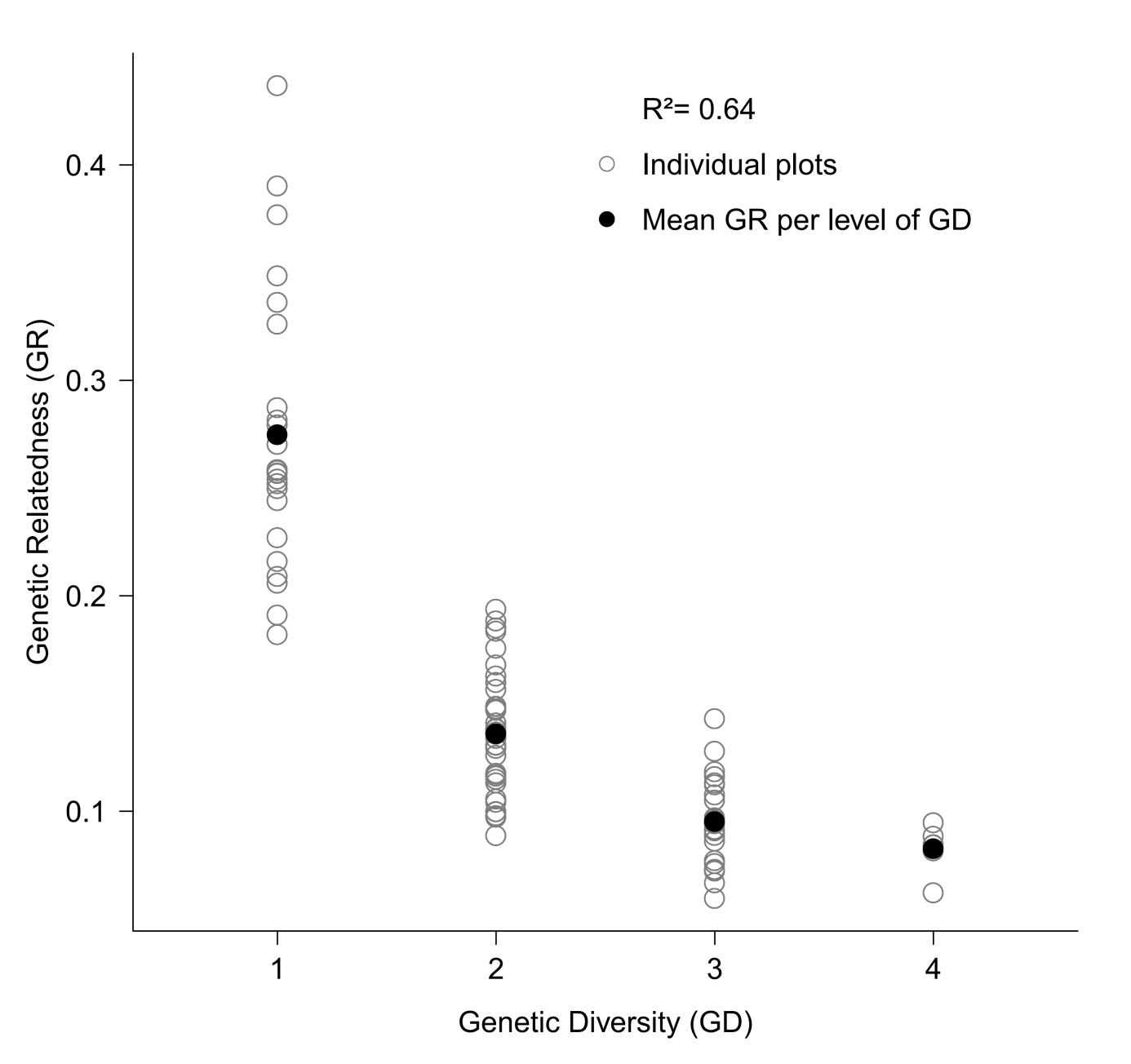

Supplement: Figure S2 — Effect of the number of half-sib families per plot (Genetic Diversity, GD) on the mean genetic relatedness among oak seedlings within plots (Genetic relatedness, GR). Open circles represent individual plots (n = 90); filed circles represent mean genetic relatedness per level of genetic diversity. (DOCX) [file pone.0044247.s002.docx]
