# Supplementary material for: Genetic Diversity Increases Insect Herbivory on Oak Saplings
Source: PLoS One. 2012 Aug 28;7(8):e44247. doi: 10.1371/journal.pone.0044247 (PMC3429418; doi:10.1371/journal.pone.0044247)
Supplement: Figure S3 — Negative selection effect of genetic diversity on exophagous herbivores. (DOCX) [file pone.0044247.s003.docx]

**Supplementary information**

**Figure SI4: Negative selection effect of genetic diversity on exophagous herbivores.**


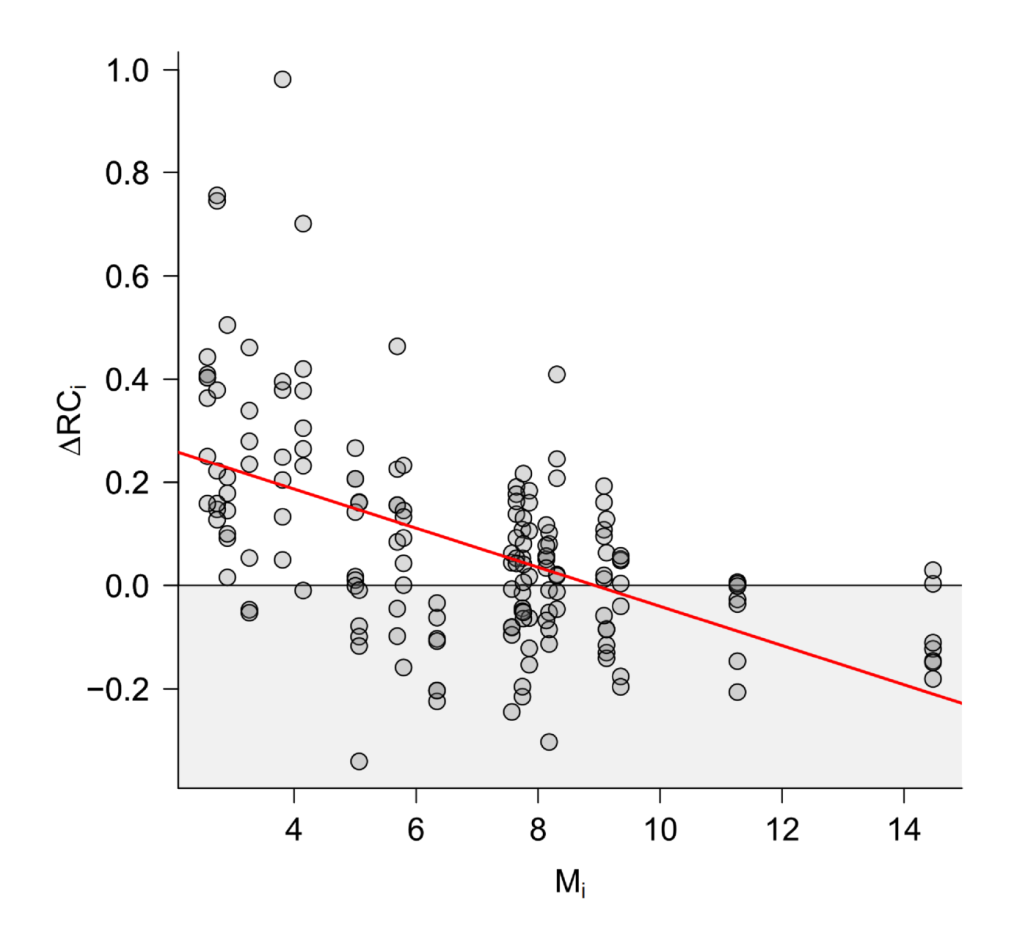


Relationship between damage in monoculture (M_i_) and deviation of the observed level of damage in mixtures from the expected level of damage (*ΔRC_i_ = RF*_O_*_i_ - RF*_E_*_i_* ). For ΔRC_i_, each data point represents the deviation between observed damage averaged across all saplings from a given family in a given plot within a given block and expected damage in the corresponding monoculture from the same block. It is plotted against mean damage on the corresponding monoculture. As each family *‘i’* was replicated six times in each block (3 folds in 2-families mixtures, 2 in 3-families mixtures and once in the 4-families mixture), each individual value of M_i_ is plotted against seven different values of ΔRC_i_ for each block. Finally, 168 data points are plotted (6 blocks × 4 families × 7 mixed plots per family per block). In the shaded area, the level of damage on the corresponding families was lower in mixtures than expected from the corresponding monocultures. The red line represents the ordinary least squares regression between M_i_ and ΔRC_i_.
