# Supplementary material for: Genetic Diversity Increases Insect Herbivory on Oak Saplings
Source: PLoS One. 2012 Aug 28;7(8):e44247. doi: 10.1371/journal.pone.0044247 (PMC3429418; doi:10.1371/journal.pone.0044247)

**Figure S4.** **Effects of genetic diversity on oak height heterogeneity within plots**.

Each dot represents the mean difference (± SE) between the 75^th^ percentile and the median (50^th^ percentile) of sapling heights distribution within plots. This difference indicates how far taller trees were apparent to herbivores within plots.


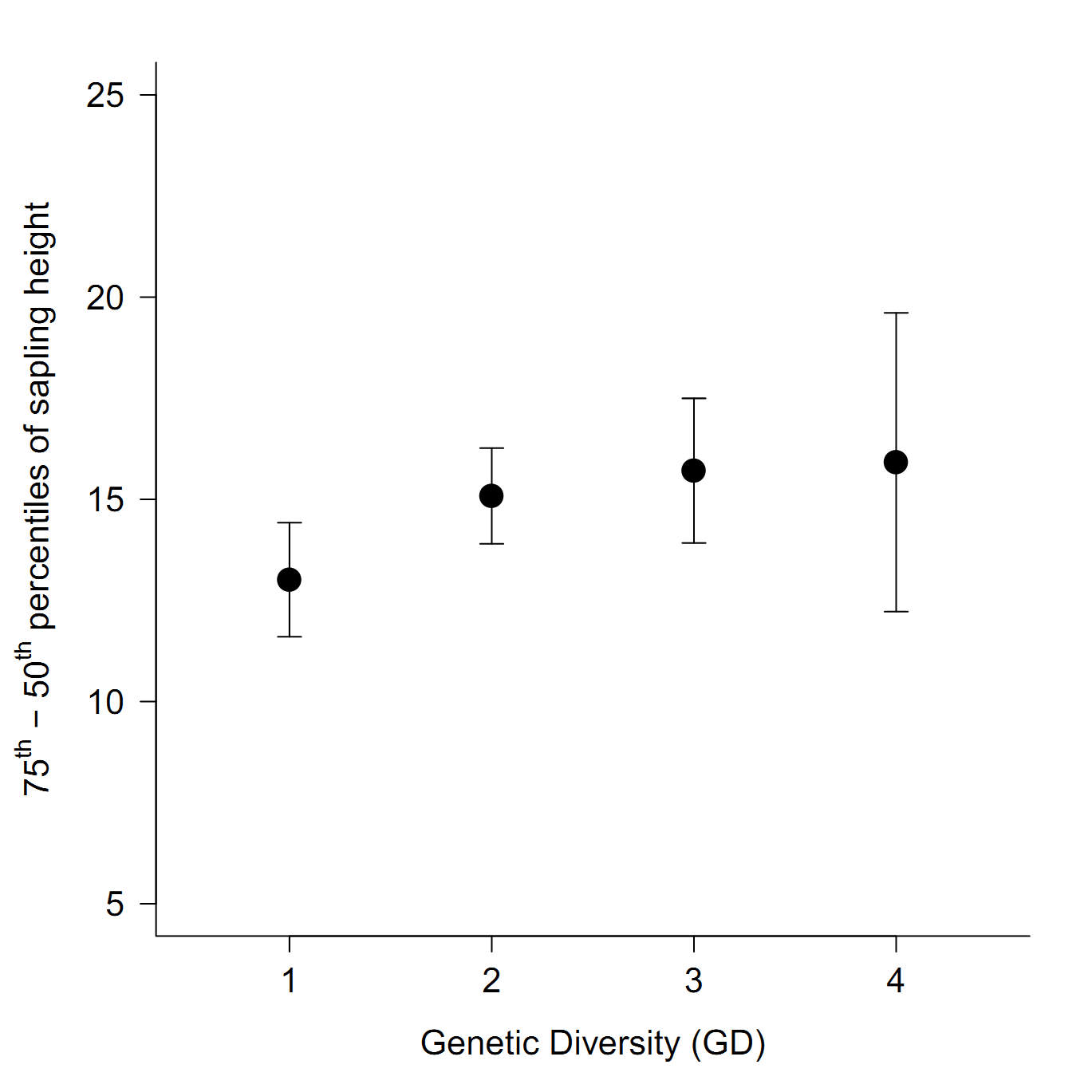

Supplement: Figure S4 — Effects of genetic diversity on oak height heterogeneity within plots. Each dot represents the mean difference (± SE) between the 75th percentile and the median (50th percentile) of sapling heights distribution within plots. This difference indicates how far taller trees were apparent to herbivores within plots. (DOCX) [file pone.0044247.s004.docx]
