# Supplementary material for: Genetic Diversity Increases Insect Herbivory on Oak Saplings
Source: PLoS One. 2012 Aug 28;7(8):e44247. doi: 10.1371/journal.pone.0044247 (PMC3429418; doi:10.1371/journal.pone.0044247)
Supplement: Table S1 — Summary of genetic data describing the genetic structure of the population used to constructed experimental plots. (DOCX) [file pone.0044247.s005.docx]

**Table SI1: Summary of genetic data describing the genetic structure of the population used to constructed experimental plots.**

|  | | **MT1** | **MT2** | **MT3** | **MT4** | **Total** |
| --- | --- | --- | --- | --- | --- | --- |
| **Missing genotypes^1^** | | 8 | 15 | 7 | 17 | 30 |
| **Mismatch^2^** | | 7 | 3 | 2 | 5 | 12 |
| **Number of descent successfully genotyped (out of 270)** | | 255 | 252 | 261 | 248 | 768 |
| **Observed heterozygoty** | | 0.659 | 0.683 | 0.725 | 0.693 | 0.690 |
| **% loci correctly genotyped** | | 0.998 | 0.993 | 0.999 | 0.996 | 0.997 |
| **Number of alleles per locus** | **PIE223** | 9 | 9 | 9 | 10 | 10 |
|  | **PIE227** | 6 | 4 | 6 | 8 | 8 |
|  | **PIE215** | 7 | 7 | 7 | 7 | 7 |
|  | **PIE258** | 13 | 13 | 17 | 17 | 19 |
|  | **PIE267** | 13 | 9 | 11 | 12 | 13 |
|  | **PIE271** | 7 | 8 | 9 | 8 | 10 |
|  | **PIE239** | 10 | 8 | 5 | 8 | 11 |
|  | **PIE102** | 7 | 8 | 7 | 9 | 10 |
|  | **PIE242** | 12 | 11 | 12 | 10 | 13 |
|  | **PIE243** | 6 | 5 | 5 | 6 | 6 |
|  | **PIE152** | 11 | 10 | 13 | 13 | 14 |

^1^ Dead or unamplified individuals

^2^ Mismatch between the genotype of the sapling and its mother’s one. Individuals with a mismatch were not included in the analyses.
